# The Combined Effect of Visual Stimulus Complexity and Semantic Content on Audiovisual Associative Equivalence Learning

**DOI:** 10.1002/brb3.70902

**Published:** 2025-09-21

**Authors:** Kálmán Tót, Noémi Harcsa‐Pintér, Adél Papp, Balázs Bodosi, Attila Nagy, Gabriella Eördegh

**Affiliations:** ^1^ Department of Physiology, Albert Szent‐Györgyi Medical School University of Szeged Szeged Hungary; ^2^ Department of Theoretical Health Sciences and Health Management, Faculty of Health Sciences and Social Studies University of Szeged Szeged Hungary

**Keywords:** associative learning | audiovisual | complexity | human | psychophysics | semantic content

## Abstract

**Background:**

The Rutgers Acquired Equivalence Test (RAET) is an associative learning task that requires participants to learn pairs of visual stimuli and then recall and generalize these associations. To further explore this cognitive task, we developed three audiovisual learning tests with the same structure as the original RAET.

**Methods:**

Each audiovisual test applied the same four distinct auditory antecedents but differed in visual consequents in complexity and semantic content, that is, cartoon faces (SoundFace), colored fish (SoundFish), and geometric shapes (SoundPolygon), respectively. The present study investigated the effect of these different visual stimuli on performance in audiovisual associative equivalence learning. Learning performance was assessed across three phases: acquisition, retrieval, and generalization. A total of 52 participants (25 females, 27 males, mean age = 25.88 ± 10.28 years) completed the tasks. Statistical analyses, including Friedman's ANOVA and Wilcoxon matched‐pairs tests with Bonferroni correction, were applied to evaluate differences in performance across the tests.

**Results:**

Participants consistently performed significantly (*p* < 0.01) better and responded faster in learning, retrieval, and generalization phases of the SoundFace test compared to the SoundFish and SoundPolygon tests, which did not significantly differ from each other. Additionally, a semantic association task confirmed that face and fish stimuli were significantly (*p* < 0.01) richer in semantic content than polygons, yet only face stimuli significantly facilitated audiovisual learning outcomes.

**Conclusion:**

These results suggest that the semantic content of visual stimuli—which could influence their verbalizability—is not sufficient on its own to enhance performance in audiovisual associative learning. Additionally, the number and variety of different features in visual stimulus sets (such as faces, fish, or polygons) may also significantly influence performance in audiovisual equivalence learning.

## Graphical abstract

This study compares audiovisual associative learning using faces, fish, and polygons as visual stimuli with identical auditory antecedents. Results show better performance with faces, with no significant difference between fish (despite semantic content) and polygon stimuli. These findings highlight the importance of feature richness and verbalizability in audiovisual learning.

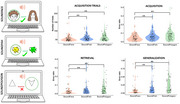

## 1 Introduction

Acquired equivalence learning is a form of associative learning where subjects come to understand that two or more stimuli are interconnected and regarded as equivalent due to their association with a common outcome or response. This understanding allows for the extrapolation of learned equivalences to new situations (generalization). Myers et al. (2003) developed a computer‐based visual learning test known as the Rutgers Acquired Equivalence Test (RAET) to explore this learning mechanism. The core principle of RAET involves learning pairs of visual stimuli (cartoon faces and fish) through trial‐and‐error. The test unfolds in two primary phases: acquisition and test. In the acquisition phase, participants progressively learn pairs of stimuli, receiving feedback on the accuracy of their responses. This process also establishes equivalence among stimuli leading to the same outcome. During the test phase, feedback is withheld, challenging participants to recall the associations they have learned (retrieval). Moreover, the test evaluates the ability to deduce unshown associations based on established equivalences, thus testing for predictable links (transfer or generalization).

The advantage of the test is that the underlying neural structures involved in its two phases are relatively well known, which enables targeted investigations in different disorders where the given structures are affected (Bódi et al. 2009; Eördegh et al. 2020; Giricz et al. 2021; Kéri et al. 2005; Myers et al. 2003; Pertich et al. 2020; Rosu et al. 2022). Previous studies have indicated that the acquisition phase primarily depends on the integrity of the basal ganglia–frontal lobe loops, whereas the test phase mainly depends on the integrity of the hippocampal region (de Araujo Sanchez and Zeithamova 2023; Gogtay et al. 2006; Larsen and Luna 2015; Moustafa et al. 2010; Myers et al. 2003; Persson et al. 2014; Porter et al. 2015; Shohamy and Wagner 2008).

Stimuli from our natural, everyday environment are typically multimodal, so integrating information from various independent sensory modalities to create a unified representation, known as multisensory integration, can be more informative for detecting and evaluating a biologically significant event than relying on a single environmental stimulus (Gibney et al. 2017; Marucci et al. 2021; Meredith and Stein 1983; Stein et al. 2014). Earlier research has established that brain structures crucial for acquired equivalence learning, such as the basal ganglia and hippocampi, are also involved in multisensory information processing and integration (Bates and Wolbers 2014; Chudler et al. 1995; Lee et al. 2017; Nagy et al. 2006; Ravassard et al. 2013; Schwarz et al. 1984).

Therefore, to explore multimodal processing in acquired equivalence learning, our research team developed an audiovisual test (SoundFace) modeled after the structure of the original RAET. The only variation in this version was the use of distinct sounds as antecedents and cartoon faces as consequents (Eördegh et al. 2019). The inclusion of a cross‐modal test (audiovisual) allows for a more ecologically valid investigation of how sensory modalities interact in real‐world contexts, where individuals are often exposed to multimodal stimuli. By pairing auditory stimuli with visual stimuli of varying complexities, this study aims to better understand how multisensory integration impacts learning and generalization in acquired equivalence tasks Supplementary Material.

The novelty and straightforward aspect of the present study is the application of visual consequent stimulus sets of varying complexity (faces, fish, or polygons), semantic content, and verbalizability in audiovisual learning, using the same auditory stimulus set as antecedents. We retained the auditory stimuli from the SoundFace test as antecedents but altered the consequent stimuli, replacing the cartoon faces with four differently colored fish (all identical in size and shape, SoundFish) or with simple two‐dimensional blank geometric shapes (SoundPolygon). These visual sets can be characterized by varying numbers of features. Additionally, it seems to be a different challenge to verbalize the faces, the fish, and the polygons due to their differing semantic contents.

In a previous study, where we compared the SoundFace and SoundPolygon tests pairwise, we observed that a reduction in semantic meaning and complexity of the visual consequent stimuli adversely affected performance in both the acquisition phase and the test phase (involving retrieval and generalization) (Tót et al. 2022). In the present study, we expanded this approach to include the SoundFish test, aiming to refine our understanding of the role of visual stimulus complexity and semantic meaning in audiovisual equivalence learning. We ask whether performances in the SoundFish test would fall between that of SoundFace and SoundPolygon or more closely resemble one or the other.

The theoretical question guiding this study is how visual complexity, semantic content, and verbalizability of visual consequent stimuli in an audiovisual context influence associative learning, retrieval, and generalization. The present study focuses on semantic content; therefore, we additionally conducted an analysis of the semantic content of the visual stimuli sets. To address these questions, we compared performance across all three audiovisual tests in a within‐subjects design.

This research has broader implications for understanding associative learning in cognitive architecture, particularly in how multisensory integration affects learning performance. The findings may offer insights into clinical applications by informing interventions for disorders that impact associative learning and multisensory integration (e.g., neurological conditions). Moreover, this study has potential educational applications, particularly in designing effective learning environments that incorporate multimodal stimuli to enhance engagement and retention.

## 2 Methods

### 2.1 Participants

A priori power analysis was conducted using G*Power 3.1.9.7 to determine the required sample size for detecting a medium effect (*f* = 0.25) in a repeated‐measures ANOVA with three conditions. The analysis, assuming *α* = 0.05, power = 0.90, and a correlation of 0.5 between repeated measures, indicated that a minimum of 36 participants was required. In this study, we present the results of 52 healthy adults, ensuring adequate statistical power. The sample comprised 25 females and 27 males, with an average age of 25.88 ± 10.28 years, ranging from 18 to 58 years. Three participants were excluded from the analysis as they did not meet the inclusion criteria. Participants were recruited on a voluntary basis and received no compensation. They were informed that they were free to withdraw at any point without any negative consequence. Before the study commenced, participants were briefed on its background, objectives, and procedures, and they provided their consent by signing an informed consent form. Participants were asked whether they had any diseases, conditions, and addictions or were taking any medications to ensure they were free of psychiatric or neurological disorders. Color blindness was ruled out using Ishihara plates. Auditory stimuli were presented to participants individually before each testing session to ensure they could hear, recognize, and distinguish them correctly.

### 2.2 The Applied Audiovisual Tests

The tests were run on Lenovo ThinkBook 15 IIL laptops (Lenovo, China) equipped with circumaural headphones (Sennheiser HD439, Sennheiser, Germany) for delivering auditory stimuli. The sessions took place in a quiet room, ensuring that participants could engage with the tasks without interruptions. Participants were tested one at a time to prevent distractions. They were allowed to complete the tasks at their own pace without any imposed time constraints or the pressure to respond quickly, thus avoiding the impact of time pressure. The order of the three tests (SoundFace, SoundFish, and SoundPolygon) was pseudorandomized across participants to minimize both carry‐over effects and fatigue. The total duration of the experiment was approximately 15–20 min. Given the length of the experiment, short breaks were provided between tasks to minimize fatigue effects. The study design accounted for the potential impact of fatigue by allowing participants to rest and recover between tasks.

**FIGURE 1:**
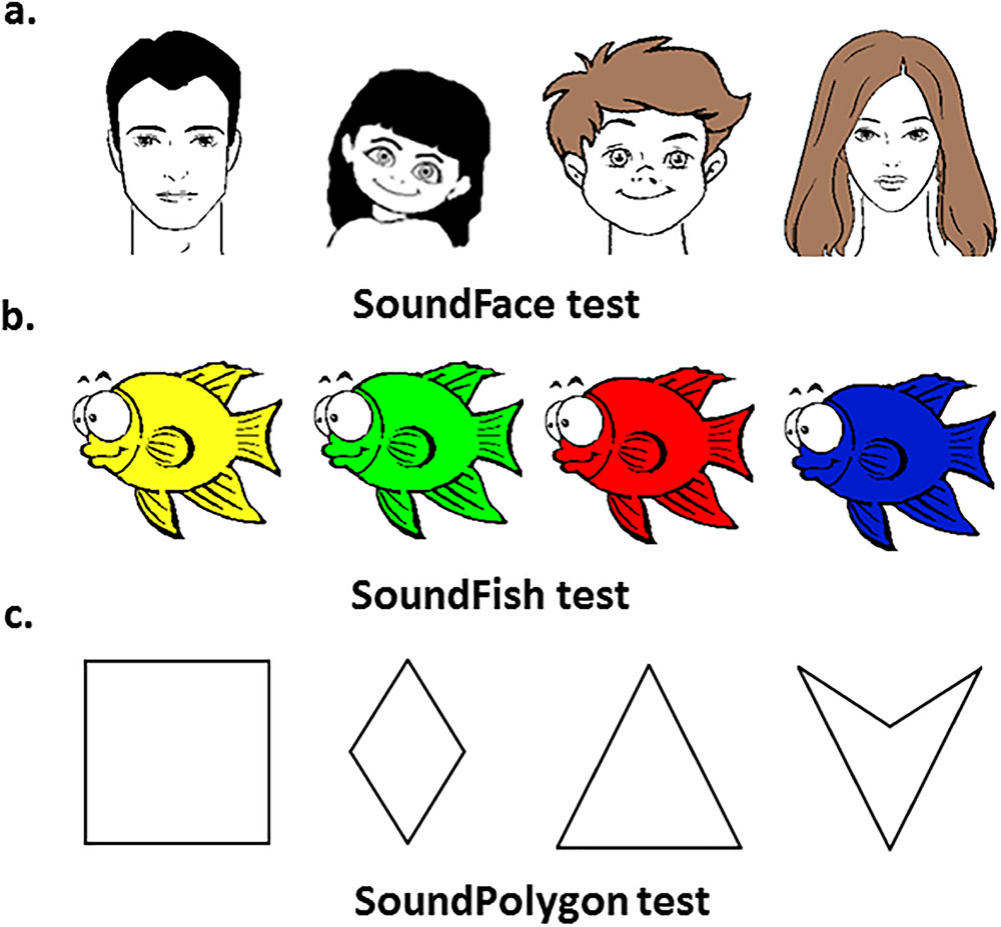
The applied visual consequents in the three audiovisual tests: (a) SoundFace test ‐ cartoon faces distinguished by gender, age, and hair color, (b) SoundFish test ‐ fish of identical shape differentiated only by color, (c) SoundPolygon test ‐ geometric shapes

All audiovisual tests followed a uniform design, modeled after the RAET (Myers et al. 2003), with modifications authorized by the original authors. The test was translated into Hungarian and programmed in Assembly language for Windows. Its primary objective is to facilitate the learning of associations between four antecedents (A1, A2, B1, and B2) and four consequents (X1, X2, Y1, and Y2) via trial‐and‐error. In each test, the antecedents were distinct sounds: a cat's meow, a woman's voice, a vehicle's noise, and a guitar chord. The same four auditory stimuli were used as antecedents across all three tests, but their pairings with visual stimuli were randomized and counterbalanced to prevent familiarity effects or fixed associations. For the SoundFace test, the consequents were cartoon faces representing a man, a woman, a boy, and a girl, distinguished by three binary semantic features: gender (male vs. female), age (young vs. adult), and hair color (brown vs. black) (Eördegh et al. 2019). In the SoundFish test, the consequents were fish of identical shape and size, differentiated solely by color (green, yellow, blue, or red). The SoundPolygon test featured various two‐dimensional blank geometric shapes (square, triangle, rhombus, and concave deltoid), with only a limited number of distinguishing features (Figure 1) (Tót et al. 2022).

**FIGURE 2:**
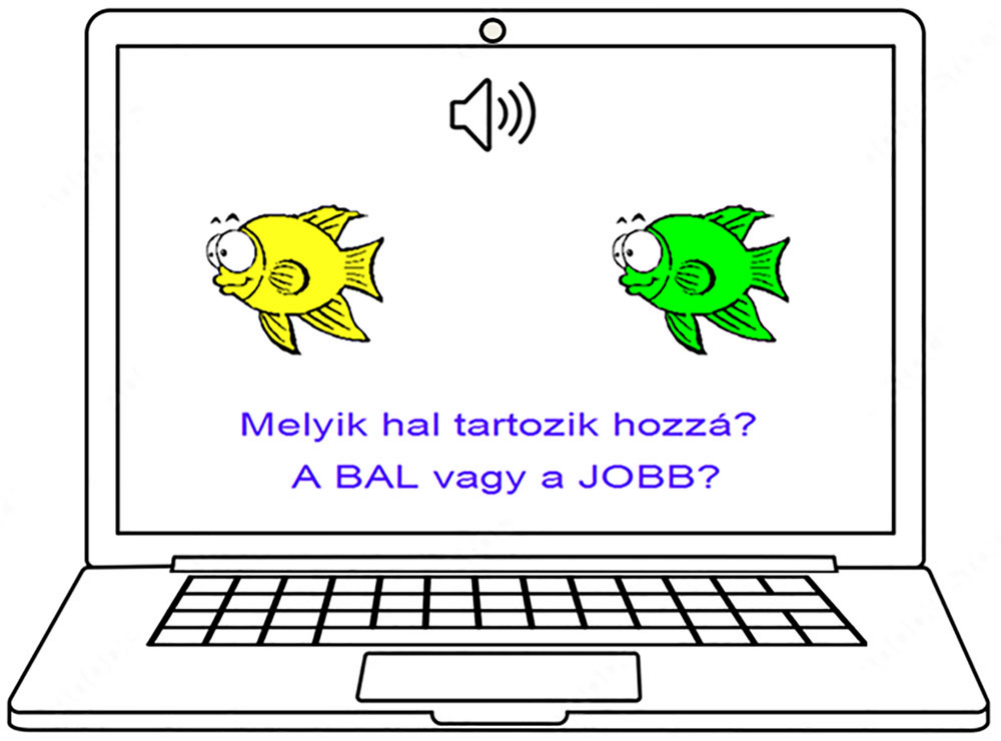
A trial during the acquisition phase of the SoundFish test. In every trial, participants simultaneously experience a sound (antecedent) and view two fish (consequents) positioned on the left and right sides of the screen. Participants then decide which of the two fish corresponds to the antecedent sound by pressing either the “left” or “right” button. The format of the trials remains consistent across other tests, with only the visual stimuli changing. The text displayed on the screen translates to English: “Which fish belongs to it? The LEFT or the RIGHT?”.

The tests consist of two primary phases: the acquisition and the test. During the acquisition phase, participants learn the pairs of stimuli through feedback provided by the software. In each trial, they are presented with a sound (antecedent) and two visual consequents displayed on the left and right sides of the screen (Figure 2). Participants must infer that consequent is associated with the presented antecedent by pressing either the “left” or “right” button. The antecedent stimuli were matched for duration (1500 ms) and loudness (60 dB), ensuring consistency across all trials. The visual consequents remained visible until a response was done. This controlled presentation method helped minimize any variability in auditory stimuli that could influence the associative learning process. Immediate feedback is provided to indicate the accuracy of their response: a green check mark alongside the word “correct” (“Helyes!”) for correct answers and a red X with the word “incorrect!” (“Helytelen!”) for incorrect answers. The side positioning of visual stimuli (left/right) and the assignment of auditory antecedents to visual consequents were randomized and counterbalanced across participants to prevent systematic pairing effects.

Participants gradually learn the pairs of stimuli. After the introduction of each new stimulus pair, a specific number of consecutive correct responses (4, 6, 8, 10, and 12 for each successive new association, respectively) are required to advance further in the test and complete the acquisition phase; thus, the total number of trials depends on the efficiency of the learning process. Initially, participants familiarize themselves with the first two pairs of stimuli (A1: X1 and B1: Y1). Following this step, new antecedents are introduced while retaining the same consequents (A2: X1 and B2: Y1), leading to the formation of equivalence between these pairs (A1 = A2 and B1 = B2) because they are associated with identical consequents. After establishing this equivalence, new consequents are presented (A1: X2 and B1: Y2), assigning additional consequents to A1 and B1. At this juncture, participants have learned six out of the possible eight pairs, thereby concluding the acquisition phase.

**FIGURE 3:**
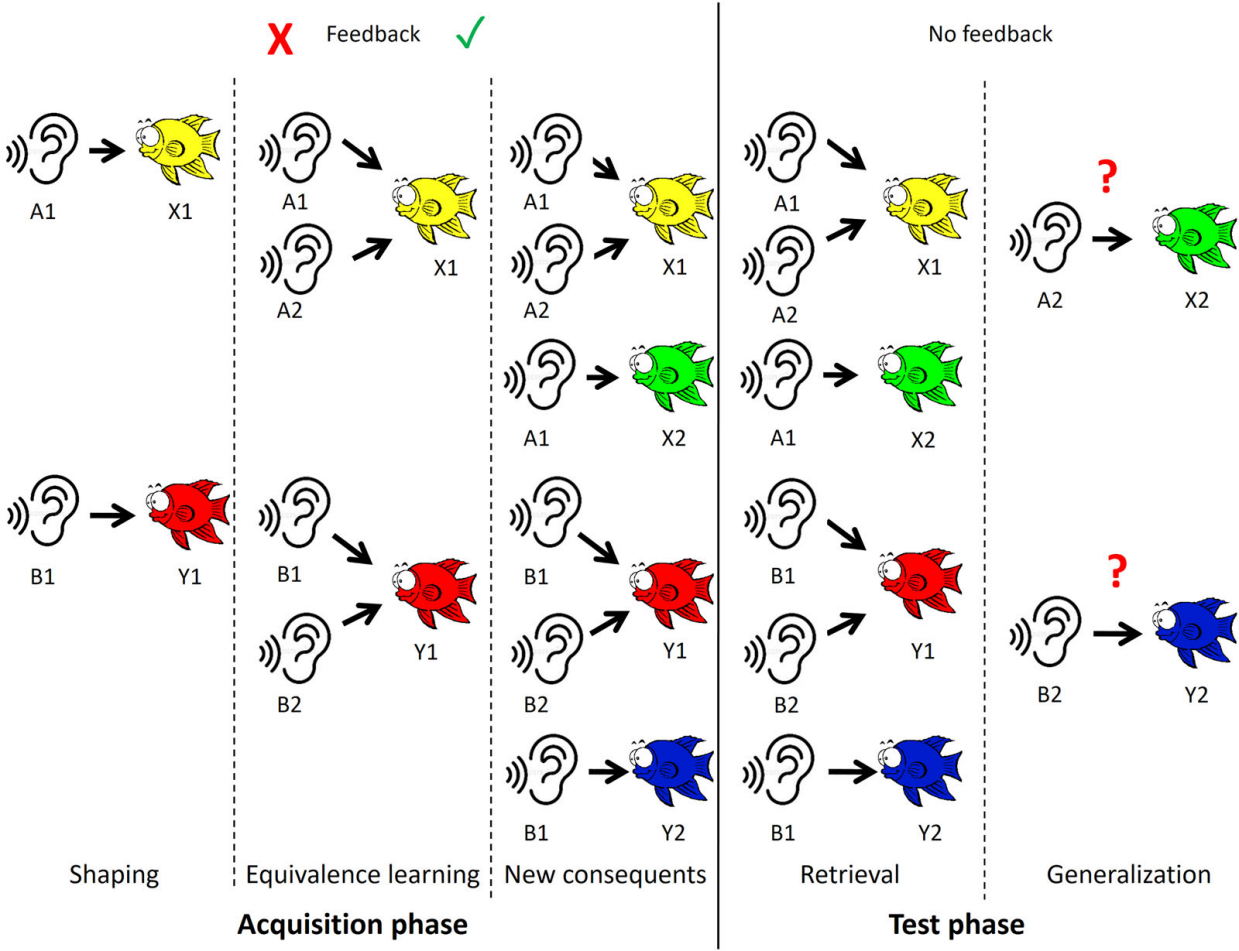
Overview of the SoundFish Test. The test is divided into two primary phases: acquisition and testing. During the acquisition phase, participants learn the pairs of stimuli through feedback provided by the software. In the testing phase, the feedback of the correctness of responses is discontinued. Participants are expected to recall the associations they have learned and extend this acquired knowledge of equivalence to new pairs of stimuli that have not yet been presented.

During the test phase, participants no longer receive feedback regarding the accuracy of their answers. They are required to recall the associations they have learned (retrieval) while also being introduced to the two remaining pairs (A2: X2 and B2: Y2) for generalization. Successful equivalence learning should enable participants to apply their knowledge to these new stimuli. Participants were not preinformed about the introduction of new stimulus pairs. Unlike the acquisition phase, where the number of trials varied, the test phase had a fixed number of trials: 36 for retrieval and 12 for generalization. The sequence of these trial types was randomly arranged by the software. The procedural steps and overall structure of the tests are depicted in Figure 3.

### 2.3 Quantifying the Semantic Content of the Visual Stimulus Sets

To quantify the semantic content of the visual stimuli used in the three audiovisual learning tests, an additional semantic association task was conducted with 44 participants. A priori power analysis (G*Power 3.1.9.7) indicated that a minimum of 43 participants was required to detect a medium effect size (*f* = 0.25) with *α* = 0.05 and power = 0.90.

Each participant viewed 12 visual stimuli (four faces, four fish, and four geometric shapes), presented individually in randomized order. Each stimulus appeared on the screen for 10 s, followed by a 2‐s blank screen and a 0.1‐s red “X” fixation cross marking the transition to the next trial. The total duration of this semantic association task was approximately 2.4 min (12 stimuli × 12 s). Given the short duration of the task and the randomization of stimuli presentation, fatigue effects were minimized.

Participants were instructed to verbally list as many words or concepts as they could that came to mind during stimulus presentation. We intended to analyze in the present study the number of connected associations to different visual stimuli. Thus, the participants were explicitly asked to avoid describing physical features (e.g., “long hair” or “child”) and instead focus on associative or conceptual content (e.g., “school” and “underwater”). Any descriptive responses were excluded during data cleaning. For each group (Face, Fish, and Polygon), repeated associations were counted only once per participant, and the total number of unique associations was calculated.

Although one group showed mild deviation from normality, a repeated‐measures ANOVA was used due to its robustness in moderately sized samples. The analysis revealed a significant main effect of stimulus group on semantic richness (*p* < 0.001). Post hoc comparisons were conducted using paired *t*‐tests or Wilcoxon signed‐rank tests, based on the normality of the difference scores (assessed via the Shapiro–Wilk test). Specifically, *t*‐tests were used for the Face–Fish and Face–Polygon comparisons, and a Wilcoxon signed‐rank test was used for the Fish–Polygon comparison.

### 2.4 Data Analysis

The evaluation of test performances utilized four key measures: The number of trials needed to learn the associations during the acquisition phase (NAT), acquisition error ratio (AER), retrieval error ratio (RER), and generalization error ratio (GER). These error ratios were derived by dividing the number of incorrect responses by the total trial count for each respective phase.

Additionally, reaction times (RTs) were precisely measured in milliseconds, with an average calculated for each phase (acquisition, retrieval, and generalization). RT refers to the interval from when the stimuli are presented to when the participant responds. Data points exceeding three standard deviations from the mean were omitted from the analysis. The number of excluded data points was minimal, and the effect of their exclusion on the overall results was negligible.

For statistical analysis, Statistica 14.0.0.15 (TIBCO Software Inc.) was applied. The Shapiro–Wilk test was used to assess the normality of data distribution. The data distribution was not normal; thus, Friedman's ANOVA was applied to contrast behavioral performance measures across the three tests. Significant differences indicated by this test warranted further pairwise comparisons using the Wilcoxon matched‐pairs test. Given the multiple pairwise comparisons conducted for each of the seven dependent variables, we applied a Bonferroni correction to control for the risk of Type I error. The corrected significance threshold was set at 0.017 (0.05 divided by the three pairwise comparisons). Effect sizes (*r*) for the Wilcoxon matched‐pairs tests were calculated as *r* = *Z*/√*N*, where *Z* is the test statistic and *N* is the number of observations. A post hoc power analysis was conducted using G*Power for all Wilcoxon matched‐pairs tests, based on the observed effect sizes (Cohen's *d_z_*), *α* = 0.05, and the valid sample sizes per comparison.

## 3 Results

**FIGURE 4:**
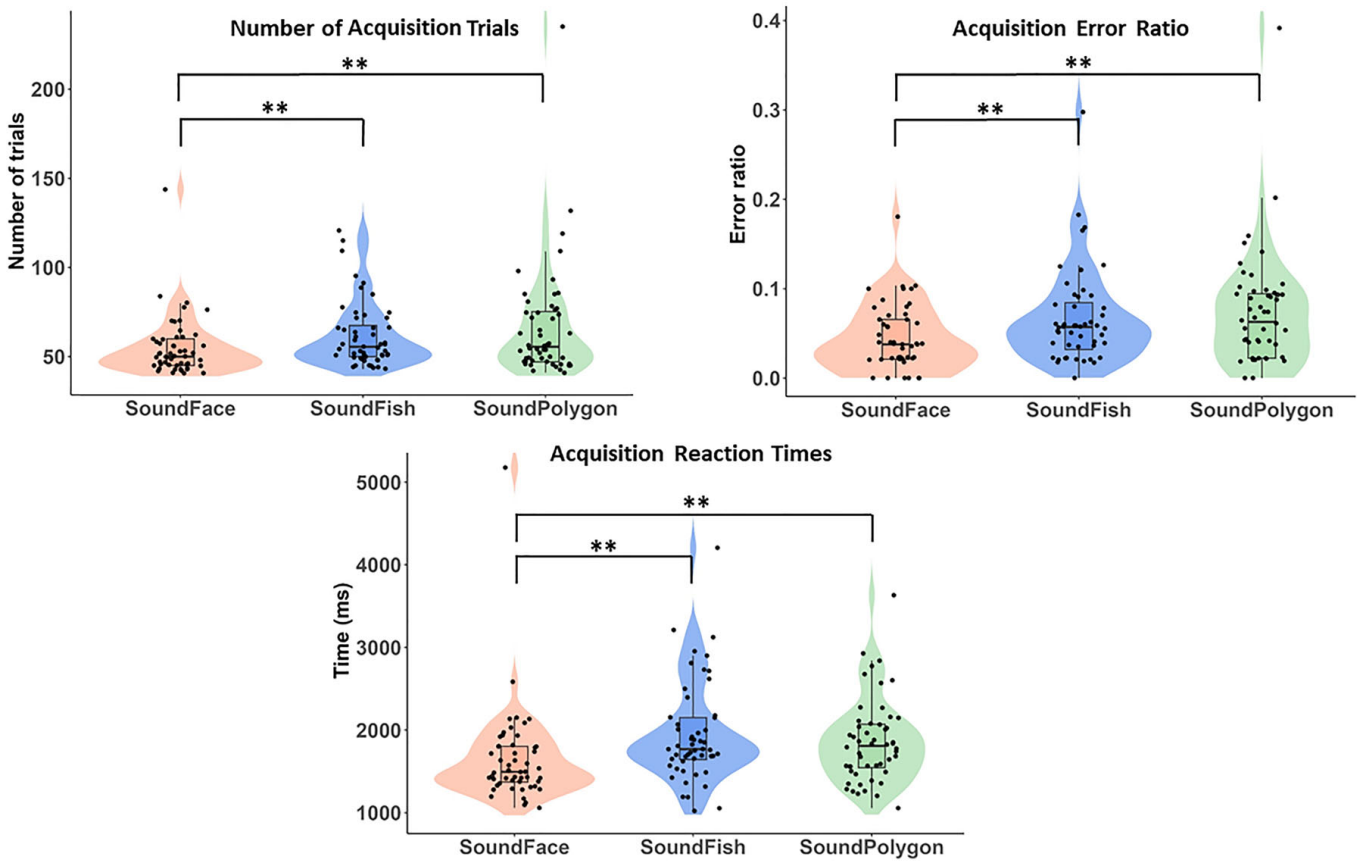
Learning performances and reaction times during the acquisition phase. The lower margin of the boxes indicates the 25th percentile, the upper margin the 75th percentile, whereas the line within the boxes marks the median. The error bars (whiskers) extend to the most extreme data points within 1.5 × IQR from the quartiles. The dots represent individual data points. Asterisks indicate statistically significant differences (*p* < 0.01).

**FIGURE 5:**
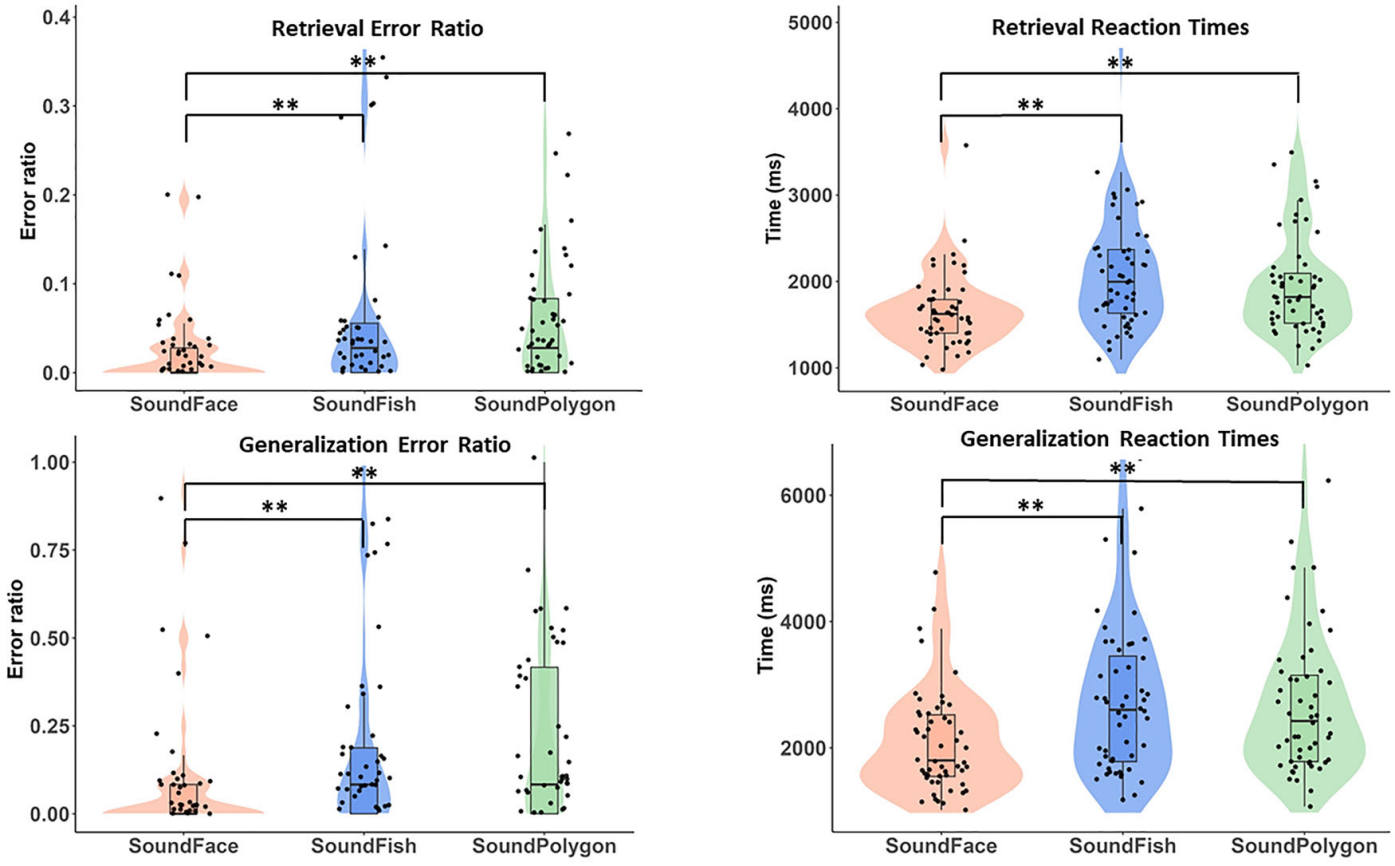
Retrieval and generalization performance and reaction times in the test phase. Otherwise, the conventions are the same as in Figure 4.

In the present study, we report the performances of 52 healthy and adult participants. Friedman's ANOVA revealed significant differences (*p* < 0.05) across all measured parameters among the three audiovisual tests (SoundFace, SoundPolygon, and SoundFish): NAT (*χ* ^2^(2) = 10.935; *p* = 0.004), AER (*χ* ^2^(2) = 7.686; *p* = 0.021), RER (*χ* ^2^(2) = 12.132; *p* = 0.002), GER (*χ* ^2^(2) = 12.418; *p* = 0.002), acquisition RT (*χ* ^2^(2) = 20.462; *p* < 0.001), retrieval RT (*χ* ^2^(2) = 21.385; *p* < 0.001), and generalization RT (*χ* ^2^(2) = 18.115; *p* < 0.001). Descriptive statistics are shown in Table 1 and Figures 4 and 5. Pairwise comparisons between the tests are presented separately according to different phases of the task (acquisition or test). The significant differences are highlighted in the figures.

**TABLE 1:** Descriptive statistics of performance measures across the three audiovisual tests.

**SoundFace**			
Parameter	Median	Lower quartile (Q1)	Upper quartile (Q3)
NAT	50	45	60
AER	0.038	0.021	0.066
Acquisition RT	1302.98	1133.94	1491.64
RER	0.000	0.000	0.028
Retrieval RT	1369.56	1194.46	1531.36
GER	0.000	0.000	0.083
Generalization RT	1554.13	1231.50	2061.58
**SoundFish**			
NAT	55.5	50	69
AER	0.057	0.029	0.087
Acquisition RT	1497.66	1264.05	1916.35
RER	0.028	0.000	0.056
Retrieval RT	1661.30	1349.03	2045.23
GER	0.083	0.000	0.208
Generalization RT	2257.25	1549.33	3331.60
**SoundPolygon**			
NAT	55.5	47	75.5
AER	0.063	0.022	0.095
Acquisition RT	1499.14	1260.27	1782.30
RER	0.028	0.000	0.083
Retrieval RT	1531.31	1277.91	1848.47
GER	0.083	0.000	0.417
Generalization RT	2131.57	1699.08	2720.17

*Note*: All reaction time (RT) measures are in milliseconds (ms).

Abbreviations: AER, acquisition error ratio; GER, generalization error ratio; RER, retrieval error ratio; RT, reaction time.

### 3.1 Acquisition Phase

In the SoundFace test, participants required significantly fewer trials to complete the acquisition phase (NAT) compared to both the SoundFish (*Z* = 2.573; *p* = 0.010; *r* = 0.357; 1 − *β* = 0.796) and the SoundPolygon tests (*Z* = 2.973; *p* = 0.003; *r* = 0.420; 1 − *β* = 0.888). Similarly, the AER was also significantly lower in the SoundFace test compared to the SoundFish (*Z* = 2.805; *p* = 0.005; *r* = 0.389; 1 − *β* = 0.855) and the SoundPolygon (*Z* = 2.896; *p* = 0.004; *r* = 0.410; 1 − *β* = 0.874) tests. Not only was performance better in SoundFace, but RTs were also shorter than those in SoundFish (*Z* = 4.499; *p* < 0.001; *r* = 0.612; 1 − *β* = 0.995) and SoundPolygon (*Z* = 3.810; *p* < 0.001; *r* = 0.518; 1 − *β* = 0.967).

In contrast, no significant differences were observed in NAT (*Z* = 0.688; *p* = 0.492; *r* = 0.100; 1 − *β* = 0.162), AER (*Z* = 0.555; *p* = 0.579; *r* = 0.078; 1 − *β* = 0.133), or acquisition RT (*Z* = 0.279; *p* = 0.780; *r* = 0.038; 1 − *β* = 0.166) between the SoundFish and SoundPolygon tests. Results of the acquisition phase are illustrated in Figure 4.

### 3.2 Test Phase

In the test phase, participants performed significantly better when tested with the SoundFace test than with the other two tests. Specifically, error ratios were lower in SoundFace compared to SoundFish (RER: *Z* = 2.855; *p* = 0.004; *r* = 0.490; 1 − *β* = 0.860; GER: *Z* = 2.875; *p* = 0.004; *r* = 0.479; 1 − *β* = 0.866) and SoundPolygon (RER: *Z* = 2.991; *p* = 0.003; *r* = 0.473; 1 − *β* = 0.890; GER: *Z* = 2.749; *p* = 0.006; *r* = 0.458; 1 − *β* = 0.838). Moreover, the SoundFace test featured significantly shorter RTs, surpassing both the SoundFish (retrieval: *Z* = 4.473; *p* < 0.001; *r* = 0.609; 1 − *β* = 0.996, generalization: *Z* = 4.827; *p* < 0.001; *r* = 0.669; 1 − *β* = 0.987) and SoundPolygon (retrieval: *Z* = 3.328; *p* < 0.001; *r* = 0.453; 1 − *β* = 0.958; generalization: *Z* = 3.855; *p* < 0.001; *r* = 0.530; 1 − *β* = 0.966) tests in efficiency.

Between the SoundFish and SoundPolygon tests, the differences were not statistically significant in terms of RER (*Z* = 0.252; *p* = 0.801; *r* = 0.045; 1 − *β* = 0.080) and GER (*Z* = 0.350; *p* = 0.726; *r* = 0.055; 1 − *β* = 0.094) during the test phase. Likewise, there were no significant differences in retrieval (*Z* = 1.253; *p* = 0.210; *r* = 0.170; 1 − *β* = 0.319) and generalization RTs (*Z* = 0.500; *p* = 0.919; *r* = 0.070; 1 − *β* = 0.063) between these tests. The results of the test phase are illustrated in Figure 5.

### 3.3 Semantic Content Analysis of the Visual Stimulus Sets

To quantify the semantic content of the visual stimuli, the total number of unique meaningful associations per stimulus group was measured (see Section 2). Descriptive statistics indicated that fish and face stimuli get the same amount of associations (fish: *M* = 12.14, SD = 3.45; faces: *M* = 12.11, SD = 3.84), but they were higher than those of the polygons (*M* = 10.02, SD = 3.04). A repeated‐measures ANOVA using Wilks’ Lambda revealed a significant main effect of stimulus group on semantic richness, Wilks’ *Λ* = 0.0637, *F* (3, 29) = 142.06, *p* < 0.001. Post hoc analyses showed no significant difference between the Face and Fish groups, *t* (43) = −0.04, *p* = 0.967, *d* = 0.01, but both groups elicited significantly more associations than the Polygon group (Face > Polygon: *t* (43) = 3.97, *p* < 0.001, *d* = 0.60; Fish > Polygon: *Z* = 3.48, *p* < 0.001, *r* = 0.53).

## 4 Discussion

To our knowledge, this is the first study to systematically compare audiovisual associative equivalence learning across three visual stimulus sets varying in complexity, semantic content, verbalizability, and number of different features of stimuli within one stimulus set. The three learning tests used identical auditory antecedents (animal, human, vehicle, and instrument sounds), whereas the applied visual consequents were different: cartoon faces, colored fish, and geometric shapes.

The present results confirmed our previous findings that audiovisual acquired equivalence relied on pairwise comparisons between the SoundFace and SoundPolygon tests in a different participant sample. Both results showed the priority of the faces in audiovisual learning (Tót et al. 2022). Furthermore, the application of three different visual stimulus sets in the present study aimed to refine and allow the assessment of the effect of visual stimulus characteristics on audiovisual associative learning. In the SoundFace test, visual stimuli varied in multiple properties and contained many features. In the SoundFish test, all stimuli were identical in shape and size, with plenty of features, but varied only by color, and SoundPolygon visual features were fewer than in the other two sets, but the differences among polygon stimuli were more than those of the fish stimuli.

The comparison of the three audiovisual tests revealed that participants’ performance was best in the SoundFace in all phases of the task (acquisition, retrieval, and generalization) compared to SoundFish and SoundPolygon, with no significant differences between the latter two. RTs also followed this, being faster in SoundFace, whereas there were no significant differences between the SoundFish and SoundPolygon tests. These findings suggest that the cartoon face stimuli facilitated both more accurate and efficient learning, whereas the fish and polygon visual stimuli resulted in similar and comparatively weaker performance and slower responses. The semantic association task confirmed that both face and fish stimuli were richer in semantic content than polygons, yet only face stimuli resulted in significantly better learning performance. This finding suggests that semantic content alone is not enough to explain improved multisensory associative learning.

Although we observed no significant differences between the SoundFish and SoundPolygon conditions, it is important to note that the statistical power of our analyses for several dependent measures was relatively low (with power values ranging from 0.063 to 0.319) for NAT, AER, RER, and GER. This suggests that the absence of significant differences may be due to insufficient power to detect small effects. Future studies with larger sample sizes and advanced statistical methods, such as equivalence testing or Bayesian analysis, would be beneficial in providing more robust conclusions regarding the role of visual feature complexity in audiovisual associative learning.

Face stimuli offered not only more semantic associations but also greater feature variability than fish and polygon stimuli. Previous studies indicate that when stimuli or their characteristics are more easily verbalized, learning becomes more efficient and quicker (Arslan et al. 2020; Zettersten and Lupyan 2020). The superior performance with face stimuli can be attributed to their social and semantic relevance, as well as the specialized neural processing they receive, such as activation in the fusiform face area (Elson et al. 2022; Hadjikhani et al. 2009; Tong et al. 2000; Tót et al. 2022; Zhao et al. 2019). In contrast, fish and polygon stimuli, while richer in semantic content for the fish, were likely less engaging due to their limited visual complexity and fewer distinguishing features, leading to weaker performance (Dickins et al. 1993; Fields et al. 2012).

The lack of significant performance differences between SoundFish and SoundPolygon is remarkable, given that the semantic association task confirmed higher semantic content for fish stimuli. One interpretation is that, although semantic meaning and verbalizability are important, the number and distinctiveness of visual features might also play a critical role. The face stimuli varied across three salient semantic categories (gender, age, and hair color), the polygons across two (angles and side lengths), and the fish across only one (color). However, when considering visual complexity, the cartoon faces had numerous discriminable and verbalizable features (e.g., eye shape, hair style, and face outline), whereas the fish were identical in all but color. The polygons, although lower in semantic content, still differed along more than one visual feature, which could compensate for the more semantic meaning of the fish stimulus set. Thus, it is possible that the fish and polygon stimuli were perceived as similarly undifferentiated, resulting in comparable levels of performance. Consequently, detecting differences between stimuli with many features requires less attention, whereas distinguishing between stimuli with fewer features (one or two) poses a more challenging task (Biederman 1987; Nairne 1990; Palmer et al. 2005; Zeigenfuse and Lee 2010). Because the same auditory stimulus set was applied in all three learning tests, this seems to be the most probable explanation of our findings, but other factors could also influence performance in the three audiovisual tests that differ only in visual stimulus sets.

The ease with which participants could link visual and auditory stimuli based on preexisting associations could also contribute to better performances in the SoundFace test. Forming connections between familiar everyday sounds (e.g., a meow, a voice, a guitar chord, or a vehicle sound) and cartoon faces may have been more intuitive and semantically congruent than forming such associations with fish or abstract polygons. Thus, the face stimuli may have benefitted not only from feature richness and verbalizability but also from being more easily integrated with their auditory antecedents. To check this issue in more detail, the application of reduced auditory stimuli with minimal semantic meaning and the same visual stimulus sets seems to be appropriate.

Another possible explanation for the differences in learning strategies is that participants relied on explicit learning rules for more verbalizable stimuli (like faces), leading to faster and more accurate learning (Ashby and Ell 2001; Maddox and Ashby 2004; McLaren 2018; Smith et al. 2012; Wasserman et al. 2023). In the SoundFace test, features, such as age, gender, and hair color, could serve as meaningful anchors for explicit categorization. By contrast, SoundFish and SoundPolygon stimuli, with fewer verbalizable features, likely promoted slower, more gradual implicit learning. These memory systems operate concurrently yet in a competitive manner during feedback‐based category learning (Ashby and Maddox 2005, 2011; Astley 1998). The transition from explicit to implicit learning strategies has also been previously demonstrated in probabilistic learning (Poldrack and Gabrieli 2001) and virtual navigation tasks (Iaria et al. 2003). Although this interpretation is plausible, it should be considered cautiously given that strategy use was not directly measured in this study. Future work using post‐task interviews or confidence ratings could provide direct evidence to distinguish between explicit and implicit learning processes.

One limitation of the present study is that order of the three tasks was pseudorandomized, but we did not formally assess whether performance in later tasks was systematically affected by earlier ones, as the small sample size per task sequence (approximately nine participants) prevented a robust analysis. In some of our previous studies, we investigated the effect of the order in the case of two tests, but there was no effect on the performances (Eördegh et al. 2022; Tót et al. 2022). However, in this study with three tests, the effect was not tested statistically; therefore, order, fatigue, or practice effects may have influenced performance. Future studies with larger sample sizes may be better equipped to address this. Additionally, repeated exposure may introduce practice or strategy transfer effects across tasks, which should also be considered a potential source of variability in the results.

Other limitation of the present study is that the sample covers a wide age range (18–58 years), which may introduce age‐related variance in associative and multisensory learning. Although the majority of participants were students, with a median age of 22, future research will aim to specifically analyze the effects of age and gender on performance. These factors were not directly addressed in the current analysis but should be considered potential sources of variability in our findings. Additionally, the current design could not fully isolate the effects of semantic richness and visual feature variability, as these factors were intertwined in the stimulus sets. Moreover, the semantic association task excluded descriptions of physical features (e.g., “long hair” or “child”) to focus on associative or conceptual content. Although this approach aimed to capture semantic richness through deeper associations, we have to acknowledge that excluding physical descriptors in the semantic association task may omit an important aspect of how people naturally categorize and verbalize visual stimuli, especially for stimuli like faces, which are highly verbalizable in terms of physical features (e.g., gender, age, and hair color). This decision may have affected the construct of semantic richness and the operationalization of verbalizability, potentially underestimating the role of easily verbalizable features. Future research should aim to quantify both feature complexity and semantic richness separately to better understand how each contributes to audiovisual associative learning.

## 5 Conclusion

In summary, our findings suggest that the semantic richness and the complexity of the consequent visual stimulus sets are not enough to fully explain the different performances in audiovisual associative learning with the same antecedent auditory stimulus set. Although face and fish stimulus sets were equally rich in semantic content, only the applied face stimuli consistently led to better learning and faster responses compared to the polygon stimulus set in audiovisual equivalence learning. These results point to the combined influence of multiple factors, that is, the number and distinctiveness of visual features and the ease of verbalizing visual stimulus characteristics, which could support the formation of meaningful links between auditory and visual modalities.

## Author Contributions

**Kálmán Tót**: writing – original draft, visualization, writing – review and editing, formal analysis, data curation, investigation. **Noémi Harcsa‐Pintér**: investigation, writing – review and editing. **Adél Papp**: investigation, writing – review and editing, formal analysis. **Balázs Bodosi**: software, writing – review and editing. **Attila Nagy**: conceptualization, writing – original draft, writing – review and editing, resources, supervision, methodology, funding acquisition. **Gabriella Eördegh**: conceptualization, methodology, writing – review and editing, project administration, supervision.

## Ethics Statement

The study's protocol adhered to the principles of the Declaration of Helsinki in every aspect and received approval from the Regional Research Ethics Committee for medical research at the University of Szeged, Hungary (27/2020‐SZTE).

## Consent

All participants provided written informed consent.

## Conflicts of Interest

The authors declare no conflicts of interest.

## Peer Review

The peer review history for this article is available at https://publons.com/publon/10.1002/brb3.70902

## Supporting information




**Supplementary Materials**: brb370902‐sup‐0001‐SuppMat.xlsx

## Data Availability

The datasets generated and/or analyzed during the current study are available from the corresponding author on reasonable request.
